# Democratic societies defeat (COVID-19) disasters by boosting shared knowledge

**DOI:** 10.1007/s11299-021-00278-0

**Published:** 2021-05-12

**Authors:** Laura Martignon, Shabnam Mousavi, Joachim Engel

**Affiliations:** 1 Ludwigsburg University of Education, Ludwigsburg, Germany; 2 Max Planck Institute for Human Development, Berlin, Germany

**Keywords:** COVID-19, Corona virus, Numeracy, Information representation, Policy making, Risk literacy, Democracy, Transparency

## Abstract

Preparing people for dealing with hazards, diseases and disasters requires teaching them statistics, and ideally doing so by means of good representation formats in a dynamic fashion. Translating these dynamics to simple communication is what governments need from scientists.

In an appeal to German residents^1^ to observe Covid-19 related restrictions on March 19th, 2020 Angela Merkel began the conclusion of her message by upholding democratic values: *Wir sind eine Demokratie. Wir leben nicht von Zwang, sondern von geteiltem Wissen und Mitwirkung. Dies ist eine historische Aufgabe und sie ist nur gemeinsam zu bewältigen*. She declared, “We are a democracy. We do not live by constraint, but by shared knowledge and participation. This is a historic task and it can only be accomplished together.^2^” It is hard to contest that effective participation in the social dynamics of a democracy requires clear understanding of facts and rules by the people. The interesting question for policymakers is how to functionally generate this shared knowledge.

Scientists can help. The trick to effective communication lies in representing information in accessible ways, by saying, for instance, “one in 10” instead of 10%. Using absolute in place of relative numbers, representing information in appealing graphic forms, and summarizing most relevant facts in “fact boxes” are some of the methods developed and advocated for increasing transparency in communication between stakeholders such as health providers and patients^3^. Once information is easily grasped, the related instructions and rules become transparent, and in turn people’s reasoning for following (or rejecting) them will also gain clarity.

But it is not enough to produce clear and accessible information alone. Wrong information can be presented in very clear ways as well. What is crucial is to design forms of education that can instill the capacity for both absorbing information and debunking misinformation. Endowing societies with such competencies will play out profoundly in times of crisis, when the quality of information upon which actions ensue is particularly consequential.

In the Middle Ages, people viewed crises such as the Black Death (estimated to have killed at least one third of the population in Europe) as calamities^4^ fallen from heavens to punish sinners. Accordingly, they would reach out to spiritual and religion authorities for guidance. Later on, following scientists and other authorities became more popular, as exemplified a century ago in the case of the Spanish Flu^5^ (estimated to have killed up to 50 million worldwide). Today, people prefer less to follow others and more to decide for themselves. In this mode, everyone today seems to turn to the Internet where information is abundant but often incomplete and unclear, and sometimes outright wrong. The only way to immune citizens from making their choices and evaluating governmental decisions based on “bad” information is to equip everyone with the competency to “read” the numbers correctly and with tools to assess the veracity of contents. Like any skill, numeracy (numerical literacy) is better acquired sooner than later, and best internalized during childhood.

We write this piece to promote the design and implementation of educational tools and techniques that prepare a society for dealing with all forms of adverse events. A great and inspiring example of such tools at work appeared in the *Washington Post* on March 14, 2020^6^ entitled *Why outbreaks like coronavirus spread exponentially, and how to “flatten the curve.”* Graphics reporter Harry Stevens^7^ used the data from Johns Hopkins University Center for Systems Science and Engineering^8^ to communicate the dynamics of the spread of the virus. In this article, Stevens starts with explaining the term “exponential” both verbally and visually with a graph before going on to use a line of five moving dots—representing individuals—that contract a disease by coming in contact with each other in a sequence. Next, he expands the demo to many such dots, and generates the resulting change over time for different degrees of social distancing. The *Washington Post* made this “story” available for free and in all major languages, leading to its being widely used throughout the world, including repeatedly on German television. Scientific platforms such as Germany’s Robert Koch Institute also provided sophisticated versions of simulations that reproduce the spread of infectious disease for the benefit of researchers^9^. Although their sophistication varies depending on the target audience, the fact remains that simulations make for “good” information representations.

A key point to notice here is that hazardous situations are dynamic by nature. That is, the way we interact with them has an impact on their final outcome. The science that studies the structure of multiple future outcomes is statistics^10^. Thus, preparing people for dealing with hazards starts by teaching statistics, and ideally doing so in a dynamic fashion. Translating these dynamics to simple communication is what governments need from scientists. One month into the lockdown, the German Chancellor, a quantum chemist by education, set an example of how effective communication could be done. She first clarified the *goal* of her government, “so it doesn’t overtax our health system,” and reported the *current state* of spread as Germany is retaining adequate health services under restrictions, “now, we are at the spread rate of 1, that is one person contaminates one other.” Then, she explained *future possibilities* and what they would amount to “at a rate of 1.1, by October the capacity of our health system will be met in terms of intensive care unit (ICU) beds. At a rate of 1.2—i.e., if of every five infected persons, one of them infects two and the rest each infect one other person—the limit will be reached already in July….^11^” Besides its clarity, this representation of information has an added virtue of accountability as it makes the assessment of proposed policies easily measurable. Democratic societies thrive on transparency, and transparency is enhanced by good communication of information. To this end, we advocate active integration of numeracy modules in public education curricula.

Coronavirus pandemic has revealed an overwhelming global desire for better understanding of socio-economic situations and political decisions, manifested in big part through searches conducted online by people from all walks of life. For example, a highly visited semi-official source^12^ reporting on Covid-19 diagnostic tests stated:^13^ “Sensitivity refers to [the rate at which] the test comes out positive, if the [tested] sample contains some components of the virus, [i.e., a positive test result] for the tested person means that he has the virus.” Providing elaboration for a technical term, such as sensitivity, shows that the source is aware of non-technical readers of its contents. Unfortunately, they confound sensitivity and predictive value here as a positive test indeed does not imply necessarily that the person has contracted the virus. The high traffic on these types of information sites speaks to the public’s aptitude, and the high frequency of mistaken explanations begs for action. People’s aptitude for knowledge must be treated as an opportunity for building social capital. People’s desire for better understanding of facts and acquiring information must be mobilized and nourished by scientific means. And, importantly, sources that provide information must be checked by experts for accuracy.

It is plenty clear that people want to know about the differences between existing tests for Covid-19 and want to understand what sensitivity, diagnosticity or predictive value, and false positive rate means. They want to make sense of the predictive values that they hear on the news. In other words, people are demanding education on “basic tools”. This shows the relevance of our point that all fully official and semi-official entities—i.e., municipalities, first responders, NGOs, governmental offices—would benefit from being equipped with tools that communicate “truths” in transparent ways that can be well understood. We promote all involved parties to “construct” instruments that are elaborate and dynamic, and benefit from the tools already developed. One such tool—originally motivated by the spread of HIV—that has been used with success^14^ with school pupils uses the following scenario:

In the Märchenland redux, with princesses and mermaids, where “crown” is the test or symptom for “being a princess,” you have to estimate the sensitivity and the predictive value of “crown” when changing (with your slide rule) base rates or population size.

Here, it can be seen that, in the context of health literacy, the concepts of sensitivity and predictive value or diagnosticity can be illustrated by easy-to-grasp examples. Icon arrays and dynamic icon arrays (as used by the *Washington Post*) belong to the tools for communicating information on diseases but also on tests, their sensitivities, and predictive values, and on risk reductions.

In sum, our position is that people want to understand facts about events and the reasons behind governmental decisions. Scientists have developed effective methods for information representation. Policymakers, social institutes, and all active agents can benefit from transparent communication. In particular, scientists can produce user-friendly, absolutely transparent tools to illustrate not only diagnosticity, predictive values, risk reductions, and risk enhancements but also how to construct simple decision trees^15^.

In general, we promote active integration of skills for classifying information in the schools’ curricula as a necessary infrastructure for dealing with all trades of hazards, be it retirement savings and investments, making good health and medical treatment decisions, or taming and extinguishing natural and artificial disasters. Moreover, it is an effective strategy for building sustainable human capital from the existing public enthusiasm for acquiring information. Finally, it is necessary for the meaningful participation of citizens in democratic societies. Numeracy leads to resilience through knowledge.

## Footnotes

1 https://www.bundeskanzlerin.de/bkin-de/aktuelles/fernsehansprache-von-bundeskanzlerin-angela-merkel-1732134

2 Source: https://www.general-anzeiger-bonn.de/ga-english/news/angela-merkel-s-speech-about-the-corona-virus-in-full_aid-49639811

3 See, for example: https://www.hardingcenter.de

4 https://courses.lumenlearning.com/suny-hccc-worldhistory/chapter/the-black-death/

5 https://www.cdc.gov/flu/pandemic-resources/reconstruction-1918-virus.html

6 https://www.washingtonpost.com/graphics/2020/world/corona-simulator/

7 https://www.washingtonpost.com/people/harry-stevens/

8 Made available on github: https://github.com/CSSEGISandData/COVID-19/tree/master/csse_covid_19_data/csse_covid_19_daily_reports

9 See an advanced simulation adjustable for complexity and vaccination, here: https://www.complexity-explorables.org/slides/i-herd-you/

10 Learn about the activities of an international forum for statistical literacy here: https://iase-web.org/

11 Also see https://www.youtube.com/watch?v=22SQVZ4CeXA

12 https://www.mdr.de/nachrichten/ratgeber/wie-zuverlaessig-ist-der-coronatest-100.html

13 Sensitivität bedeutet, dass der Test positiv ausfällt, wenn eine Probe das Virus oder Bestandteile davon enthält. Für den Getesteten heißt das, er hat das Virus.

14 See https://www.eeps.com/riskicon/ developed by Tim Erickson.

15 See an article at https://www.semanticscholar.org/paper/TEACHING-AND-LEARNING-ABOUT-TREE-BASED-METHODS-FOR-Engel-Erickson/b61b6c36ac3ef584b3486000b155f2d015305940. See also: https://iase-web.org/documents/papers/sat2019/IASE2019%20Satellite%20160_MARTIGNO.pdf?1569666569. Also a website for constructing decision trees here, https://codap.concord.org/releases/latest/static/dg/en/cert/index.html#shared=35399

